# Deep sequencing of mRNA in CD24^−^ and CD24^+^ mammary carcinoma Mvt1 cell line

**DOI:** 10.1016/j.gdata.2015.06.032

**Published:** 2015-07-09

**Authors:** Ran Rostoker, Anitha D. Jayaprakash, Ravi Sachidanandam, Derek LeRoith

**Affiliations:** a Diabetes and Metabolism Clinical Research Center of Excellence, Clinical Research Institute at Rambam (CRIR), Haifa 31096, Israel; b Faculty of Medicine, Technion, Rambam Medical Center, Haifa 31096, Israel; c Girihlet Inc., 423 West 127th Street, First Floor, NY 10027, United States; d Department of Oncological Sciences, Icahn School of Medicine at Mount Sinai, NY 10029, United States; e Departments of Medicine, Endocrinology, Diabetes and Bone Disease, Icahn School of Medicine at Mount Sinai, NY 10029, United States

**Keywords:** CD24, Mvt1 cells, Cancer, MRNA-seq, Mammary

## Abstract

CD24 is an anchored cell surface marker that is highly expressed in cancer cells (Lee et al., 2009) and its expression is associated with poorer outcome of cancer patients (Kristiansen et al., 2003). Phenotype comparison between two subpopulations derived from the Mvt1 cell line, CD24^−^ cells (with no CD24 cell surface expression) and the CD24^+^ cells, identified high tumorigenic capacity for the CD24^+^ cells. In order to reveal the transcripts that support the CD24^+^ aggressive and invasive phenotype we compared the gene profiles of these two subpopulations. mRNA profiles of CD24^−^ and CD24^+^ cells were generated by deep sequencing, in triplicate, using an Illumina HiSeq 2500. Here we provide a detailed description of the mRNA-seq analysis from our recent study (Rostoker et al., 2015). The mRNA-seq data have been deposited in the NCBI GEO database (accession number GSE68746).

**Table:** 

Specifications	
Organism/cell line/tissue	*Mus musculus*/epithelial mammary carcinoma Mvt1 cell line
Sex	Female cell line
Sequencer or array type	Illumina HiSeq 2500
Data format Raw data:	FASTQ files
Experimental factors	CD24^−^ vs. CD24^+^
Experimental features	RNA sequencing for mRNA expression analysis in CD24^−^ and CD24^+^ cells
Consent	N/A
Sample source location	N/A

## 1 Direct link to deposited data

Deposited data can be found here: http://www.ncbi.nlm.nih.gov/geo/query/acc.cgi?acc=GSE68746.

## 2 Experimental design, materials and methods

Expression of CD24, an anchored cell surface marker, is associated with poorer outcome in cancer patients [1], [2]. We used mRNA-seq to identify transcripts that are differentially expressed in CD24^+^ cells compared to CD24^−^ cells [3].

### 2.1 Cell culture and cell sorting

The Mvt1 mammary cancer cells [4] were cultured in DMEM supplemented with 10% fetal bovine serum and antibiotics (penicillin:streptomycin) at 37 °C in a humidified atmosphere consisting of 5% CO_2_ and 95% air.

Mvt1 cells were cell surface stained for CD24 expression and sorted into two separate groups of cells, CD24^−^ and CD24^+^ cells using the FACSAria.

### 2.2 Experimental design and total RNA preparation

Total RNA was isolated and purified from 3 different plates (each group) of adherent cells grown in DMEM supplemented with 10% fetal bovine serum using the Total RNA Purification Kit (Norgen Biotek Corp) according to the manufacturer's instructions. RNA quality was assessed by the RNA analysis screentape (R6K screentape, Agilent), RNA with RIN > 9 was reverse transcribed to cDNA. cDNA libraries were prepared using 1 μg of total RNA using the TruSeq RNA Sample Preparation Kit v2 (Illumina).

### 2.3 mRNA-seq and data analysis

**Fig. 1:**
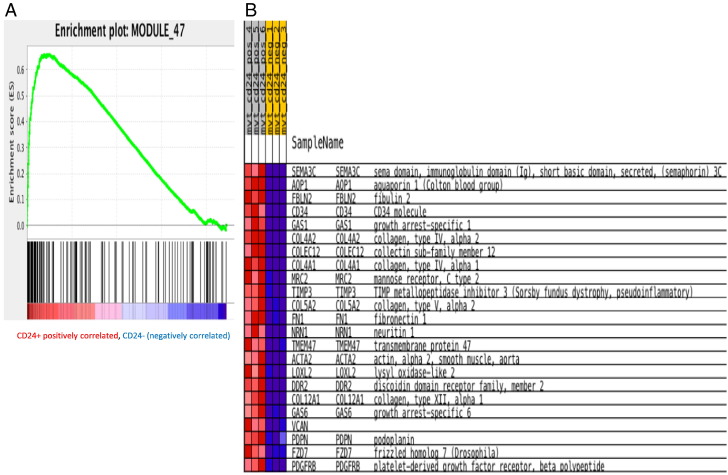
Enrichment in ECM and collagens in the tumorigenic CD24^+^ subset. A, Gene set enrichment analysis histogram of the ECM and collagen module (http://robotics.stanford.edu/~erans/cancer/modules/module_47.html). B, Heat map illustrating gene expression from the module 47.

cDNA libraries were sequenced on the Illumina HiSeq 2500 platform to obtain 51-bp single-end reads. The reads were trimmed, 2 nt on each end, to remove low quality parts, and improve mapping to the genome. The 47 nt reads that resulted were compressed by removing duplicates, but keeping track of how many times each sequence occurred in each sample in a database. The unique reads were then mapped to the mouse genome, using exact matches. This misses reads that cross exon–exon boundaries, as well as reads with errors and SNPs/mutations, but it does not have substantial impact on estimating the levels of expression of each gene. Each mapped read was then assigned annotations from the underlying genome. In case of multiple annotations (e.g. a miRNA occurring in the intron of a gene), a hierarchy based on heuristics was used to give a unique identity to each read. This was then used to identify the reads belonging to each transcript and coverage over each position on the transcript was established. This coverage is non-uniform and spiky, thus we used the median of this coverage as an estimate of the gene's expression value. In order to compare the expression in different samples, quantile normalization was used. The ratios of expression levels were then calculated to estimate the log (to base 2) of the fold-change. In order to prevent low-expressed genes from dominating the list of genes with a large fold change, we added a regularizer (10) to each value, ensuring that genes with expression around or below 10 would appear to have low fold-change [5], [6]. We did gene set enrichment analysis using the GSEA software. Of all the cancer modules that we tested our expression against, we found that module 47 was significantly up-regulated in the CD24 positive populations (Fig. 1A). Extracellular matrix proteins and collagens were enriched in this module with an FDR corrected p-value of 0.05 (Fig. 1B).

## 3 Discussion

The CD24^+^ Mvt1 subset displays highly tumorigenic and metastatic capacity compared to their CD24^−^ counterparts [3]. Taking advantage of the Illumina HiSeq 2500 we preformed transcript analysis in order to compare between the gene profiles of these two subsets. Ting DT et al. recently found that elevated expression in ECM (extracellular matrix) genes is common in different types of circulating cancer cells [7]. These results suggest that elevated expression of ECM transcripts promote cancer cell capacity to drive not only tumors at the primary site but also at distant organs.

These ECM components enable cancer cells to remodel the host tissue in order to form ‘metastatic niche’ by acting and responding to the surrounding host tissue cells [8]. In our recent study, we found that CD24^+^ Mvt-1 form rapidly mammary tumors, moreover, with the tail vein assay, our results demonstrated metastatic phenotype of these CD24^+^ cells [3]. Similar to Ting DT et al. results, our gene analysis highlighted high expression of ECM genes in this CD24^+^ aggressive subset (Fig. 1). This gene profile may reveal new mechanisms in tumorigenesis and can serve as a start point for further research with an important clinical implication for metastatic tumors.

## Conflict of interest

The authors declare no conflict of interest.
